# Exercised-based cardiac rehabilitation associates with lower all-cause mortality in patients with primary pulmonary hypertension

**DOI:** 10.3389/fspor.2023.1247615

**Published:** 2023-12-11

**Authors:** Geert Kleinnibbelink, Benjamin J. R. Buckley, Stephanie L. Harrison, Nefyn Williams, Elnara Fazio-Eynullayeva, Paula Underhill, Arie P. J. van Dijk, Gregory Y. H. Lip, Dick H. J. Thijssen

**Affiliations:** ^1^ Research Institute for Sport and Exercise Sciences, Liverpool John Moores University, Liverpool, United Kingdom; ^2^ Research Institute for Health Sciences, Departments of Physiology and Cardiology, Radboud University Medical Center, Nijmegen, Netherlands; ^3^ Liverpool Centre for Cardiovascular Science, University of Liverpool and Liverpool Heart & Chest Hospital, Liverpool, United Kingdom; ^4^ Cardiovascular and Metabolic Medicine, Institute of Life Course and Medical Sciences, University of Liverpool, Liverpool, United Kingdom; ^5^ Department of Primary Care and Mental Health, University of Liverpool, Liverpool, United Kingdom; ^6^ TriNetX LLC., Cambridge, MA, United States; ^7^ TriNetX LLC., London, United Kingdom

**Keywords:** pulmonary hypertension, primary pulmonary hypertension, exercise, cardiac rehabilitation, secondary prevention

## Abstract

**Background:**

Despite pharmacological therapies to improve outcomes of pulmonary hypertension (PH), poor long-term survival remains. Exercised-based cardiac rehabilitation (ExCR) may be an alternative strategy to improve prognosis. Therefore, using an electronic medical record (EMR) database, the objective of this study was to compare mortality between patients with primary PH with ExCR vs. propensity-matched PH patients without ExCR.

**Methods:**

The retrospective analysis was conducted on February 15, 2023 using anonymized data within TriNetX, a global federated health research network. All patients were aged ≥18 years with primary PH recorded in EMRs with at least 1-year follow-up from ExCR. Using logistic regression models, patients with PH with an EMR of ExCR were 1:1 propensity score-matched with PH patients without ExCR for age, sex, race, and comorbidities, and cardiovascular care.

**Results:**

In total, 109,736 patients with primary PH met the inclusion criteria for the control group and 784 patients with primary PH met the inclusion criteria for the ExCR cohort. Using the propensity score-matched cohorts, 1-year mortality from ExCR was proportionally lower with 13.6% (*n* = 101 of 744 patients) in the ExCR cohort compared to 23.3% (*n* = 174 of 747 patients) in the controls (OR 0.52, 95% CI 0.40–0.68).

**Conclusion:**

The present study of 1,514 patients with primary PH suggests that ExCR is associated with 48% lower odds of 1-year mortality, when compared to propensity score-matched patients without ExCR.

## 1. Introduction

Given poor long-term survival in patients with pulmonary hypertension (PH), even with optimum drug therapy (1), alternative treatment strategies are needed. Exercise-based cardiac rehabilitation (ExCR) may provide an answer. A recent European Respiratory Society statement highlighted the potential of ExCR for PH patients (2). Specifically, ExCR improves exercise capacity, quality of life and improves symptoms (NYHA classification), and currently holds a class IIa recommendation (2, 3). However, improvement of these outcomes have been observed only after relatively short time periods (3–4 months) and, more importantly, no study has examined the association between ExCR and mortality in PH patients. Therefore, adopting a retrospective observational study using a large electronic medical record (EMR) database, the objective of this study was to compare mortality between patients with primary PH with exCR vs. a propensity-matched control group of PH without ExCR.

## 2. Methods

The cohort analysis was conducted on February 15, 2023 using complete case, anonymized data within the online TriNetX platform, a global federated health research network with access to EMRs from participating academic medical centres, specialty physician practices, and community hospitals, predominantly in the United States. Eligible patients with PH were identified via Centers for Disease Control and Prevention (CDC) coding using ICD-10-CM code I27.0 “primary pulmonary hypertension”. To be eligible. For the exposure group (ExCR intervention), patients were aged ≥18 years with primary PH recorded in EMRs, prescribed ExCR within six months of PH diagnosis, and at least 12-months follow-up from ExCR. The control group included patients diagnosed with primary PH but not prescribed ExCR. EExCR in the exposure group was identified from ICD-10-CM code Z71.82 (Exercise counselling), HCPCS code S9472 (ExCR program, non-physician provider, per diem), or CPT code 1013171 (Physician or other qualified health care professional services for outpatient ExCR). ExCRPatients with secondary PH, less than 12-months follow-up, and <18 years of age were excluded. At the time of the search, 27 participating healthcare organizations had data available for patients meeting the study inclusion criteria [primary PH diagnosis, 12-month follow-up, and prescribed ExCR within 6-months of diagnosis (exposure cohort)]. Thus, following propensity score matching, the cohorts consisted of patients with primary PH, who either were referred for ExCR (exposure) or did not receive ExCR (control). 12-month mortality and rehospitalisation rates were investigated using EMR data. This study is reported as per the Strengthening the Reporting of Observational Studies in Epidemiology (STROBE) guidelines (Supplementary Data).

Baseline characteristics were compared using chi-squared tests or independent-sample *t*-tests. Using logistic regression, patients with PH with an EMR of ExCR were 1:1 propensity score-matched with PH patients without ExCR for age, sex, race, diseases of the respiratory system, disease of the circulatory system, hypertensive disease, heart failure, diabetes mellitus, chronic kidney disease, cerebrovascular disease, cardiovascular procedures (e.g., cardiography, echocardiography, cardiac catheterization, cardiac devices, electrophysiological procedures), and cardiovascular medications (e.g., beta-blockers, antiarrhythmics, diuretics, antilipemic agents, antianginals, calcium channel blockers, ACE inhibitors). These variables were chosen because they are established risk factors for mortality or were significantly different between the two cohorts. Propensity score matching was undertaken using logistic regression with “greedy nearest-neighbour matching” and a calliper of 0.1 pooled standard deviations. Logistic regression models produced odds ratios (OR) with 95% confidence intervals (CI) for mortality and hospitalisation at 1-year following PH diagnosis, comparing ExCR with propensity score-matched controls. Analyses were conducted using the TriNetX online platform and regression packages in R (4). Statistical significance was set at *p* < 0.05.

## 3. Results

In total, 109,736 and 784 patients with primary PH met the inclusion criteria for the control group and the exercise-based ExCR cohort, respectively. Compared to controls, the exercise-based ExCR cohort were older, had less females, and reported more comorbidities (Table 1). Following propensity score-matching, cohorts were well balanced for age, race, sex, comorbidities, cardiovascular medications and cardiovascular procedures (*p* > 0.05; Table 1).

**Table 1:** Baseline characteristics % (*n*)^a^ of the primary PH populations with and without CR before and after propensity score matching.

	Initial populations			Propensity score matched populations		
	Primary PH without CR (*n* = 105,055)	Primary PH with CR (*n* = 784)	*P*-value	Primary PH without CR (*n* = 757)	Primary PH with CR (*n* = 757)	*P*-value
Age (years), mean (SD)	61.4 (20.5)	62.9 (14.3)	0.049	62.8 (15.5)	62.9 (14.2)	0.859
Female	64,741 (61.6)	42.2 (321)	<0.001	318 (42.0)	321 (42.4)	0.876
Race^b^						
White	64,248 (61.2)	537 (70.6)	<0.001	546 (72.1)	537 (70.9)	0.608
Black or African American	22,437 (21.4)	138 (18.1)	0.031	142 (18.8)	138 (18.2)	0.791
Asian	1,398 (1.3)	10 (1.3)	0.968	10 (1.3)	10 (1.3)	1.0
Unknown	16,508 (15.7)	62 (8.1)	0.235	51 (6.7)	62 (8.2)	0.282
Diseases of the circulatory system	75,792 (72.1)	760 (99.9)	<0.001	755 (99.7)	756 (99.9)	0.563
Diseases of the respiratory system	49,158 (46.8)	652 (85.7)	<0.001	655 (86.5)	648 (85.6)	0.603
Hypertensive diseases	50,847 (48.4)	658 (86.5)	<0.001	663 (87.6)	654 (86.4)	0.492
Heart failure	30,774 (29.3)	605 (79.5)	<0.001	589 (77.8)	601 (79.4)	0.452
Diabetes mellitus	23,788 (22.6)	364 (47.8)	<0.001	358 (47.3)	361 (47.7)	0.877
Chronic kidney disease	17,221 (16.4)	316 (41.5)	<0.001	317 (41.9)	315 (41.6)	0.917
Cerebrovascular diseases	10,844 (10.3)	227 (29.8)	<0.001	222 (29.3)	225 (29.7)	0.866
Cardiovascular procedures^c^	45,911 (43.7)	729 (95.8)	<0.001	727 (96.0)	725 (95.8)	0.795
Cardiovascular medications^d^	61,041 (58.1)	729 (95.8)	<0.001	724 (95.6)	725 (95.8)	0.899

CR, cardiac rehabilitation and exercise programmes; SD, standard deviation.

^a^ Values are % (*n*) unless otherwise stated. Baseline characteristics were compared using a chi-squared test for categorical variables and an independent-sample *t*-test for continuous variables.

^b^ Data are taken from structured fields in the electronic medical record systems of the participating healthcare organizations, therefore, there may be regional or country-specific differences in how race categories are defined.

^c^ Cardiovascular procedures include cardiography, echocardiography, catheterization, cardiac devices, electrophysiological procedures.

^d^ Cardiovascular medications include beta-blockers, antiarrhythmics, diuretics, antilipemic agents, antianginals, calcium channel blockers, ACE inhibitors.

Using the propensity score-matched cohort, mortality at 1-year from ExCR was proportionally lower 14% (*n* = 101 of 744 patients) in the exercise-based ExCR cohort compared to 23% (*n* = 174 of 747 patients) in the controls (OR 0.52, 95% CI 0.40–0.68). There was no significant association with re-hospitalisation: 55% (*n* = 350 out of 632 patients) in the exercise-based ExCR cohort, compared to 57% (*n* = 361 out of 632 patients) in the controls (OR 0.93, 95% CI 0.75–1.16).

## 4. Discussion

We provide the first evidence that ExCR in patients with PH is associated with significantly lower 1-year mortality compared to patients who did not receive ExCR. Specifically, PH patients who were prescribed ExCR demonstrated 48% lower odds of 1-year mortality. Previous RCTs have shown improvement in exercise capacity, symptomatology, and quality of life after a relatively short-term follow-up (3–15 weeks) (5–10). However, whether these surrogate endpoints are associated with outcomes in PH has never been demonstrated (11). Our data suggests exercise-based ExCR may ultimately translate to clinically meaningful improvements in mortality. The poor long-term survival in PH, which also is demonstrated in the high 1-year mortality rate in this cohort of 23%, stresses the urgency of additional (non)-pharmacolxogical treatment to improve survival. These novel observations highlight the need for prospective research studies to confirm the utility of ExCR for patients with PH.

Some limitations warrant consideration. First, characterization of disease states and therapy were based on ICD-codes, which may vary between healthcare organisations. As such, distinction was made between primary PH (i.e., caused by disease of the pulmonary arteries) and secondary PH (i.e., secondary to other, non-vascular causes) rather than the current WHO-classification for PH into group 1–5 (3). Given the complexity of PH phenotypes, some PH patients labelled as “secondary PH” may also have a variant of primary PH. A second limitation is that we were unable to evaluate characteristics or adherence to ExCR, which limits the ability to identify “active ingredients”. Third, although we matched patients for important co-morbidities and demographic factors, residual confounding and the relatively modest sample size should be considered. Finally, we were unable to evaluate the potential effect of ExCR on morbidity, primarily due to small sample sizes of subgroups. The relatively small sample size may also explain why ExCR was not associated with lower rehospitalisation, despite the lower absolute prevalence of rehospitalisation PH patients undergoing ExCR.

## 5. Conclusion

The present study of 1,514 patients with primary PH suggests that ExCR was associated with 48% lower odds of 1-year mortality, when compared to propensity score-matched patients without ExCR. This novel data on clinical outcomes highlights the promising potential of ex CR and urgency of appropriately powered RCTs to investigate the causal effects of ex CR for patients with PH.

## Data Availability

The data analyzed in this study is subject to the following licenses/restrictions: Datasets are only available for persons aligned to TriNetX. Specific data may be requested with the corresponding author. Requests to access these datasets should be directed to Benjamin Buckley, b.j.buckley@ljmu.ac.uk.
